# Bilateral pleural empyema by *Enterobacter* infection secondary to pancreaticopleural fistula

**DOI:** 10.1002/ccr3.3425

**Published:** 2020-10-20

**Authors:** Koichi Hasegawa, Ayano Toriyama, Takashi Nomizo, Hiroko Fukata, Kenichi Goto, Yasukiyo Nakamura, Tatsuo Hamada, Naoki Kanda, Hideo Kita

**Affiliations:** ^1^ Department of Respiratory Medicine Takatsuki Red Cross Hospital Takatsuki Japan; ^2^ Department of Gastroenterology and Hepatology Takatsuki Red Cross Hospital Takatsuki Japan

**Keywords:** bacterial pleural infection, chronic pancreatitis, *Enterobacter*, pancreatic pleural effusion, pancreaticopleural fistula

## Abstract

Pleural empyema secondary to pancreaticopleural fistula can be caused by ascending infection of enteric organisms from infected pancreatic pseudocysts. This unique route of infection should be noted for appropriate empirical antibiotic therapy.

## Graphical abstract

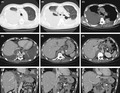

## 1 INTRODUCTION

Pancreaticopleural fistula (PPF) is a rare but well‐documented complication of chronic pancreatitis whereas bacteriologically proven cases of pleural empyema secondary to PPF are rarely reported. In this case, we identified *Enterobacter* infection in the pleural space, highlighting differences of causative microorganisms and empirical antibiotic therapy from those of community‐acquired empyema.

Pancreatic pleural effusion is an uncommon complication of chronic pancreatitis ^1^ and is caused by leakage of pancreatic secretions into the pleural space via a pancreaticopleural fistula (PPF).^2^ Disruption of a pancreatic duct or pseudocyst leads to leakage of pancreatic fluids into the subphrenic retroperitoneal space, which in turn contributes to development of a PPF. Identification of microorganisms in the pleural space is rare even in clinically diagnosed empyema secondary to PPF,^3^ and it is unknown which regimens of antibiotic therapy are necessary for this pathological condition. This report presents a case of bilateral pancreatic pleural effusion complicated by *Enterobacter* infection, which should raise awareness of possible ascending infection of enteric organisms in this pancreas‐derived pleural effusion.

## 2 CASE PRESENTATION

**Figure 1:**
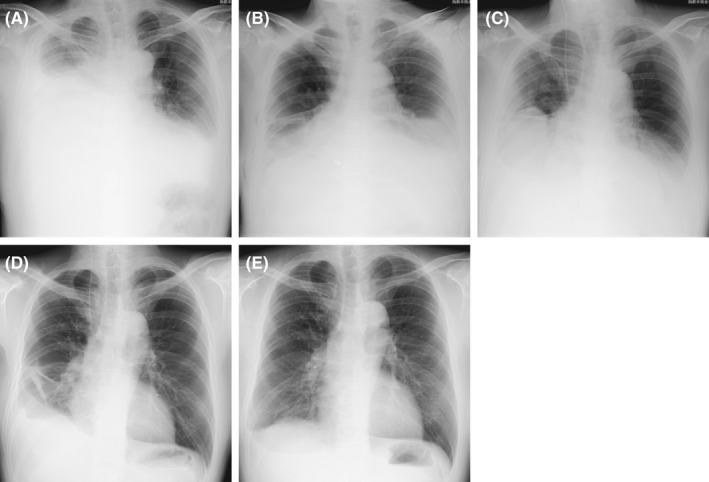
Chest radiography performed at the initial examination (A) showed a right side‐predominant bilateral opacity. Right‐sided pleural effusion was decreased following chest tube drainage on the right side (B). Right‐sided pleural effusion increased and left‐sided pleural effusion remained on day 7 (C), and a second bilateral thoracentesis procedure showed persistence of the *Enterobacter* infection. A chest radiograph obtained on day 21 (D), at which time meropenem was being administered, showed right‐sided scar‐like opacity. The opacity had nearly disappeared after 9 mo (E)

**Figure 2:**
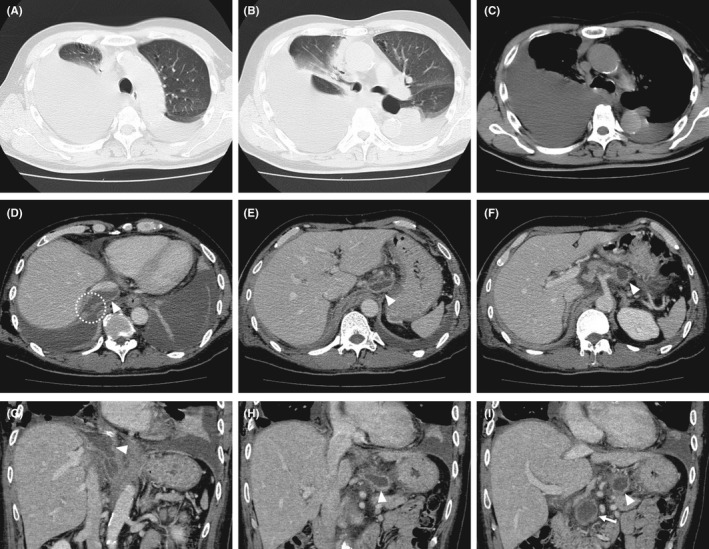
Chest computed tomography (CT) performed at the initial examination (A‐C) demonstrated right side‐predominant bilateral pleural effusion. Axial (D,E) and coronal (G,H) contrast‐enhanced abdominal CT images demonstrated a fluid accumulation in the subphrenic region (arrowhead). A fistula located between the pleural cavity and area of fluid accumulation in the subphrenic region (dotted circle) was suggested (D). Axial (F) and coronal (I) contrast‐enhanced abdominal CT images showed a pseudocyst (arrowhead) in the body of the pancreas that communicated with the fluid accumulation in the subphrenic region. Another pseudocyst (arrow) was apparent in the head of the pancreas (I)

A 50‐year‐old man came to our hospital complaining of right‐sided back pain and acute dyspnea on exertion. He was a heavy alcohol drinker with chronic pancreatitis and diabetes mellitus. Physical examination showed a tachypneic hypoxic condition, with an oxygen saturation of 93% while breathing 10 L of oxygen per minute through a simple oxygen mask. Chest auscultation revealed diminished respiratory sounds with wheezing bilaterally. No abdominal pain was noted. Chest radiography showed a homogenous opacity in the right middle to lower zone and left lower zone (Figure 1A), which was demonstrated to be bilateral pleural effusion on chest computed tomography (CT) images (Figure 2A‐C). A complete blood count showed a normal total white blood cell count (WBC) of 9500/µL with a neutrophil predominance of 78.7% and elevation of serum amylase and lipase levels to 2174 U/L and 707 U/L, respectively.

Hemorrhagic pleural effusion was obtained by a chest tube inserted into the right pleural space. WBC in the right‐sided pleural effusion was elevated to 16 800/µL with a neutrophil predominance of 92.9%. The amylase level was elevated to 86 213 U/L. Pleural effusion from the left side appeared yellowish with mild turbidity and contained an elevated amylase level of 17 583 U/L.

Contrast‐enhanced abdominal CT showed no evidence of ascites but the pancreas was mildly enlarged with slight dilation of the main pancreatic duct. There were two pseudocysts in the pancreas, and the one in the pancreatic body was extended and communicated with an accumulation of fluid in the subphrenic region (Figure 2D‐I).

**Figure 3:**
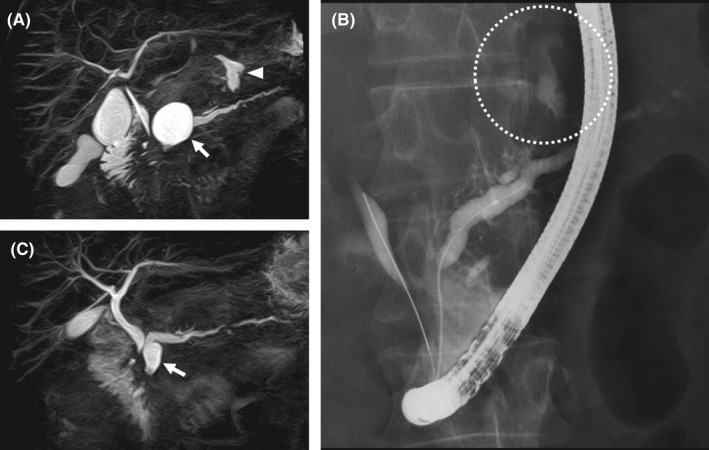
Magnetic resonance cholangiopancreatography on day 13 (A) demonstrated a pseudocyst in the body of the pancreas and fluid accumulation on the caudal side (arrowhead). Fluid accumulation in the subphrenic region was not evident at this time. The arrow indicates another pseudocyst in the head of the pancreas. Endoscopic retrograde pancreatography on day 14 (B) demonstrated communication between the main pancreatic duct and the fluid accumulation on the caudal side of the pancreatic body. Note stenosis of the main pancreatic duct and the common bile duct in their intrapancreatic portion (A,B). Magnetic resonance cholangiopancreatography at 5 mo (C) demonstrated shrinkage of the pseudocyst in the head (arrow) and disappearance of the pseudocyst in the body of the pancreas

The patient was diagnosed with bilateral pancreatic pleural effusion secondary to PPF. Right‐sided chest tube drainage was continued under total parenteral nutrition (Figure 1B), and piperacillin/tazobactam (PIPC/TAZ) was administered. Bacterial cultures of pleural fluid samples from both sides revealed *Enterobacter* species with PIPC/TAZ susceptibility. On day 6, the chest tube in the right pleural space, from which about 4000 mL of pleural effusion had been drained since admission, was removed because of reduced drainage. Repeat thoracentesis was performed on both sides of the pleural cavity on day 7 because of a slight increase in the right‐sided pleural effusion on a chest radiograph (Figure 1C). Cultures of pleural effusion samples from both sides remained positive for *Enterobacter*, which prompted a change from PIPC/TAZ to meropenem. The WBC count was slightly decreased to 8600/µL with a neutrophil predominance of 68.4% at that time. Repeat chest tube drainage was also considered but not performed because the patient's oxygenation was improved and there was multiloculated pleural effusion on the right side on ultrasonography. Magnetic resonance cholangiopancreatography performed on day 13 revealed two cysts in the head and body of the pancreas as well as a fluid collection on the caudal side of the pseudocyst in the body of the pancreas (Figure 3A). Endoscopic retrograde pancreatography on day 14 demonstrated a communication between the main pancreatic duct and the cystic lesion located on the caudal side (Figure 3B). There was no evidence of a fistula through the pleural space at that time, and an endoscopic nasal pancreatic duct tube was placed for drainage. Culture of the pancreatic fluid yielded no microorganisms.

The patient's chest pain gradually resolved on antibiotic therapy and the amount of pleural fluid also decreased (Figure 1D). The WBC count returned to the normal range, and the serum amylase level decreased to around three times the upper limit of the normal range, which was the stable level in this patient. Oral refeeding was initiated on day 19, and antibiotic therapy was continued through to day 21. On day 40, the endoscopic nasal pancreatic duct tube was removed and a pancreatic duct stent was placed for pancreatic duct stenosis in the head of the gland. Follow‐up abdominal CT performed at 3 months and magnetic resonance cholangiopancreatography at 5 months (Figure 3C) showed that both the pancreatic cyst in the body and fluid collection on the caudal side of the pancreas had disappeared. A chest radiograph at 9 months demonstrated that the opacity in the right lower zone had mostly disappeared (Figure 1E).

## 3 DISCUSSION

Pleural effusion is a complication of both acute and chronic pancreatitis, with two different types reported. The first is a low volume reactive type of pleural effusion that is generally associated with acute pancreatitis and characterized by a normal or mildly elevated amylase level of no more than 3000‐4000 IU/L in pleural fluid samples.^3^ When examined by CT, up to 50% of cases of acute pancreatitis have been shown to have pleural effusion.^4^ The second type of pleural effusion, which occurred in this patient, is direct leakage of pancreatic secretions into the pleural space via a PPF. This is usually associated with chronic pancreatitis or pancreatic pseudocysts and is characterized by rapid accumulation of a massive amount of pleural fluid, which can lead to acute respiratory failure, as in our patient. Previous case series and literature reviews have shown that about a half of diagnosed patients presented with left‐sided pleural effusion with bilateral pleural effusion observed in 14%‐16%.^3^, ^5^ In addition to direct leakage of pancreatic secretion from fistulous tract into the pleural space, transdiaphragmatic lymphatics, which join peritoneal and pleural lymphatic networks, have also been suggested to contribute to the production of pancreatic pleural effusion.^1^

Initial treatment of pancreatic pleural effusion secondary to PPF includes reduction in pancreatic stimulation by stopping oral intake, correction of fluid and electrolyte disturbance, and chest tube drainage if required for improved oxygenation. Administration of a somatostatin analog to reduce secretion of pancreatic hormones can also be considered.^6^, ^7^ Patients who are unresponsive to medical treatment including endoscopic intervention are candidates for surgical management, such as pancreaticojejunostomy and/or resection of the affected site of the gland along with the initial part of the fistula, aimed at reducing the pressure of pancreatic duct and spontaneous closure of the fistula.^8^ It is unknown whether or not antibiotic use is warranted in patients with pancreatic pleural effusion secondary to PPF, and the microbiology involved, if any, has not been elucidated.

In the present case, cultures of the bilateral pleural effusion yielded *Enterobacter*. Bacteriologically proven cases of empyema secondary to PPF are very rare,^3^ and it remains to be elucidated whether or not bacterial infection is involved in the development of PPF. Schweigert et al reported a series of six patients with PPF‐induced empyema requiring surgical intervention in whom microbiological data were not documented.^8^ A case of PPF‐induced pleural effusion with bronchopleural fistula was reported, in which *Streptococcus aureus* was isolated in both sputum and pleural fluid,^9^ implying that the bacteria originated from the lower respiratory tract.

*Enterobacter* species, which are gram‐negative bacilli of the Enterobacteriaceae family, are an important cause of nosocomial infections in the bloodstream and abdominal cavity ^10^ but are rarely isolated in pleural fluid culture.^11^ Like other enteric organisms, *Enterobacter* species can cause pancreatic pseudocyst infection or pancreatic abscess.^12^ Therefore, in this case, it is reasonable to consider the ascending infection of pleural space from an infected pancreatic pseudocyst via a PPF. This unrecognized route of bacterial entry should be noted in cases of PPF because the microbiology and empiric antibiotic therapy provided would be very different from those in community‐acquired pleural infections, in which Gram‐positive aerobic organisms such as *Streptococcus* or *Staphylococcus* are frequently isolated.^11^

Carbapenems are the preferred treatment for *Enterobacter* infections to avoid development of antibiotic resistance via the chromosomal AmpC gene. However, recent reports show that cefepime ^13^ and PIPC/TAZ ^14^ can be used instead to decrease the use of carbapenem. In the present case, PIPC/TAZ was administered initially and then changed to meropenem because of sustained bacterial growth in bilateral pleural fluid samples despite 6 days of administration. Because both the organisms isolated at the first presentation as well as those during treatment were shown to be susceptible to PIPC/TAZ in vitro, further use of PIPC/TAZ might have ultimately controlled the infection.

We could not reliably identify the species of the isolated organisms among the genus *Enterobacter* using MALDI Biotyper 3.1 (Brucker Daltonics, Billerica, MA, USA), which is based on a matrix‐assisted laser desorption ionization‐time‐of‐flight mass spectrometry (MALDI‐TOF MS) system.^15^ *E aerogenes* and *E cloacae* are the members of the genus *Enterobacter* most frequently encountered in clinical settings, although other organisms are occasionally encountered ^5^ and are reportedly difficult to identify using the MALDI‐TOF MS system.^16^ Species identification is important from a bacteriological perspective, although not always necessary for selection of antibiotics for affected patients.

In summary, we have presented a case of bilateral pancreatic pleural effusion secondary to PPF complicated by *Enterobacter* infection. Ascending infections of the pleural spaces by enteric organisms should be considered in this rare complication of chronic pancreatitis.

## CONFLICT OF INTEREST

None declared.

## AUTHOR CONTRIBUTIONS

KH: acquired and interpreted the data and wrote the final manuscript. TH: acquired and interpreted the data and approved the final manuscript. AT, TN, KG, HF, YN, NK, and HK: evaluated the data and approved the final manuscript.

## ETHICAL STATEMENT

Informed consent was obtained from the patient for publication of this case report and any accompanying images.
